# Targeting TNFR2 in Cancer: All Roads Lead to Rome

**DOI:** 10.3389/fimmu.2022.844931

**Published:** 2022-02-17

**Authors:** Jingchao Bai, Bowen Ding, Hui Li

**Affiliations:** ^1^ Department of Gastrointestinal Cancer Biology, Tianjin Medical University Cancer Institute and Hospital, Tianjin, China; ^2^ Key Laboratory of Cancer Immunology and Biotherapy, Tianjin Medical University Cancer Institute and Hospital, Tianjin, China; ^3^ National Clinical Research Center for Cancer, Tianjin Medical University Cancer Institute and Hospital, Tianjin, China; ^4^ Department of Breast Oncoplastic Surgery, Tianjin Medical University Cancer Institute and Hospital, Tianjin, China

**Keywords:** TNFR2, Treg, antagonist, agonist, tumor microenvironment

## Abstract

TNF receptor 2 (TNFR2) has become one of the best potential immune checkpoints that might be targeted, mainly because of its vital role in tumor microenvironments (TMEs). Overexpression of TNFR2 in some tumor cells and essential function in immunosuppressive cells, especially regulatory T cells (Tregs), makes blocking TNFR2 an excellent strategy in cancer treatment; however, there is evidence showing that activating TNFR2 can also inhibit tumor progression *in vivo*. In this review, we will discuss drugs that block and activate TNFR2 under clinical trials or preclinical developments up till now. Meanwhile, we summarize and explore the possible mechanisms related to them.

## Introduction

Escape from the immune system is a well-recognized feature of cancer, which has made immunotherapy the fourth most effective measure in cancer treatment after surgery, chemotherapy, radiotherapy, and targeted therapy. Immune checkpoint inhibitors have sprung up as a mainstream direction. The emergence of monoclonal antibodies (mAbs) against cytotoxic T-lymphocyte-associated antigen 4 (CTLA-4), programmed death 1 receptor (PD-1), and its ligand, PD-L1, has revolutionized the treatment landscape of cancer (1). Improved biomarkers may help to better select patients who are more likely to respond to immunotherapy and benefit new drug development. TNF receptor 2 (TNFR2) has become one of the best potential immune molecules that might be targeted mainly because of its vital role in tumor microenvironments (TMEs) (2).

TME refers to the cellular environment in the tumor site that contains non-malignant cells, tumor-infiltrating immune cells, vessels, intercellular components, metabolites, etc. (3). Recent evidence shows that TME dramatically determines the efficacy of immunotherapy (4). Regulatory T cells (Tregs) are the most extensively studied immunosuppressive cells; TNFR2 is preferentially expressed in Tregs, especially in effector Tregs, and is essential for Treg expansion and function maintenance through the classical NF-κB pathway. Meanwhile, some new molecules have also been found involved in the TNFR2 pathway.

Targeting Tregs through TNFR2 antagonists seems really promising in antitumor therapy. Interestingly, there is evidence that some TNFR2 agonists also show antitumor effects *in vivo*; some of those agonists are now in the investigational new drug (IND)-enabling phase and about to undergo clinical trials. It seems that all roads lead to Rome when targeting TNFR2 in the tumor. Here, we will review the most potential TNFR2 antagonists and agonists that are about to get into or already under clinical trials and try to explain why both blocking and activating TNFR2 can inhibit tumor cells *in vivo*. The answers might lie in the complex reactions of those non-malignant cells in the TME.

## TNFα, TNFR, and Their Signal Pathway

TNFα was first found in mice treated with bacterial endotoxin, a serological protein with necrotic antitumor activity, and hence was named tumor necrosis factor α (TNFα) (5). There are two forms of TNFα: soluble and transmembrane TNFα (sTNFα and tmTNFα). Transmembrane TNFα can be processed by TNFα-converting enzyme (TACE) to release the soluble one, and both of them are biologically active (6, 7). Many cell types are able to produce sTNFα, and the myeloid cells and activated T cells are the highest producers of this cytokine in the immune cells (8). tmTNFα is expressed on a bunch of immune cells such as macrophages and monocytes (9), dendritic cells (DCs), and natural killer (NK) cells (10). Studies have verified that tumor cells, such as in breast cancer, ovarian cancer, liver cancer, lung cancer, gastric cancer, acute lymphoblastic leukemia, and lymphoma, strongly express tmTNFα (11).

There are two receptors of TNFα: TNF receptor 1 (TNFR1) and TNFR2 (12). TNFα can bind to both TNFR1 and TNFR2; however, it is shown that sTNFα has a higher affinity for TNFR1, while tmTNFα favors TNFR2 a lot (13, 14). When binding to TNFR2, tmTNFα can mediate both forward and reverse signaling between tmTNF-α- and TNFR2-expressing cells (15, 16). These receptors can be enzymatically cleaved from the cell surface and form soluble TNFα receptors: sTNFR1 and sTNFR2. sTNFRs may inhibit TNFα bioactivity by binding to sTNFα and tmTNFα, or stabilize the trimeric structure of TNFα and prolong its bioactivity, or stimulate tmTNFα, leading to a reverse activation signal in macrophage cells, which express more tmTNFα than others (15, 17).

Upon TNFα binding to TNFR1, the cytoplasmic tail of TNFR1 recruits the adaptor protein TNFR1-associated death domain (TRADD) *via* its death domain. TRADD then interacts with TRAF2, RIPK1, or cIAP1and cIAP2 to form complex 1; and complex 1 ultimately leads to the activation of NF-κB and MAPK pathways by phosphorylating and ubiquitylating other molecules. This complex 1 pathway favors cell proliferation and survival. However, when TRADD recruits Fas-associated death domain (FADD) adaptors RIPK1 and RIPK3, complex 2 forms and leads to cell death (18, 19). In contrast to TNFR1, TNFR2 does not contain a death domain module. When TNFR2 is activated by TNFα, the intracellular domain of TNFR2 will recruit TRAF2/cIAP1/cIAP2 complexes (20, 21), resulting in the initiation of both canonical and non-canonical NF-κB activation (22–25). The PI3K/Akt pathway can also be activated reciprocally (26). Interestingly, TNFR2-dependent P38 activation varies in different cells. p38 MAPK will be activated in macrophages and murine B cells upon TNFR2 stimulation (27, 28). TNFα-induced upregulation of TNFR2 can be abrogated by p38 MAPK-specific inhibitor in CD4+ T cells (29). However, TNFR2 stimulation on TNFR2-overexpressing cancer cell lines does not result in p38 MAPK activation (30). Moreover, TNFR2 can also induce cell death indirectly by crosstalk with TNFR1 (22).

Recently, some new molecules have been found to be involved in the TNFR2 pathway. 14-3-3ϵ was recently identified as a new intracellular component of TNFR2 complexes in chondrocytes when triggered with progranulin (PGRN), and TNFR2/14-3-3ϵ signals through activating EIK-1 and suppressing NF-κB in chondrocytes (31). However, 14-3-3ϵ may play a totally different role in immunosuppressive cells in TMEs, and this needs to be proved in the future. It was verified that cardiac myocytes benefit from protection from TNFR2 activation against stress by upregulation of optic atrophy 1 (OPA1) expression, which results in improvements in mitochondrial morphology and function. This process was facilitated by p300-mediated Stat3 acetylation and Stat3/RelA interactions (32). There might be other molecules involved in the TNFR2 pathway that need to be found in the future.

## TNFR2 Is Highly Expressed in Tregs and Essential for Function Maintenance

Tregs are the most extensively studied immunosuppressive cells, and they are defined as CD4+CD25+Foxp3+ or CD4+CD25+CD127low T cells (33, 34). Current research suggests that TNFR2 is highly expressed in Tregs, especially in effector Tregs, while TNFR1 is hardly detected (35–37). The presence of high Tregs, especially TNFR2+ Tregs in the TME, is associated with an unfavorable prognosis in various types of cancers (38–40). Tregs in the peripheral blood of lung cancer patients express high levels of TNFR2, which is associated with advanced clinical stage and poor prognosis (41). This is the same situation in patients with septic shock where TNFR2+ circulating Tregs are more immunosuppressive (42).

TNFα can preferentially expand CD4+Foxp3+ Tregs *in vitro* through TNFR2 (43). Other TNFR family members, such as 4-1BB, GITR, and DR3, but not OX40, can also increase Tregs’ proliferation and survival through canonical NF-κB; TNFR2 is the most efficient among them, and the transcriptome feature of each group seems to be similar (44). Tsunoda et al. reported the generation of a new TNFR2-selective agonist TNFα mutant, termed R2gaoTNF; it could expand and activate mouse CD4+CD25+ Tregs *ex vivo*, which makes it a new candidate for Treg expansion (45). Another novel TNFR2 agonist antibody developed by Faustman can also expand highly potent Tregs (25, 46). Another novel dimeric dual-acting fusion cytokine combining IL-2 and TNFR2-selective single-chain TNF mutein (IL2-EHD2-sc-mTNFR2) showed high affinity and activation of TNFR2 and IL-2R and thus promoted superior Treg expansion (47). Paeoniflorin can ameliorate lupus nephritis in lupus-prone B6/gld mice by increasing TNFR2 expression on CD4+FoxP3+ Tregs (48).

However, some researchers think that the role of TNFα on the Tregs seems to be more complicated than it appears. On the one hand, TNFα may promote the degradation of Foxp3 by activating caspase-8 in Tregs (49) or may inhibit the expression of Foxp3 (50). On the other hand, TNFα is important for both the development and maintenance of the function of Tregs (51). This still needs to be further investigated.

There is evidence that not only tumor-infiltrating Tregs but also tumor-draining lymph nodes (TDLNs) TNFR2+ Tregs are involved in tumor progression and metastasis. Some researchers compared Tregs from tumors and matched tumor-invaded and non-invaded TDLNs, and Tregs showed conserved suppressive function in TDLN and tumor. Moreover, a common transcriptomic signature sharing by Tregs from tumors and lymph nodes was also described. TNFRSF1B transcription was alleviated obviously in Tregs in both tumor and TDLNs, regardless of lymph nodes with tumor invasion or not (52). There is also other evidence indicating that the majority of CD4+CD25+Foxp3+ Tregs are TNFR2+, and they expressed TNFR2 with the highest intensity in the TDLNs of breast cancer, up to 90.5% ± 11.3%, when compared with other CD4+ T cells, which highlights the importance of TNFR2 in Tregs (53). However, they also found that most TNFR2+CD4+ T cells were Foxp3−CD25− in the TDLNs. It seems that TNFR2 is more vital in Tregs, but we cannot ignore the majority expression of TNFR2 in Foxp3−CD4+ T cells when considering targeting TNFR2 treatment, which may influence the therapy effects or shed light on combination therapies.

## Targeting TNFR2: Blocking and Activating Drugs Under Clinical Trials or Preclinical Developments

TNFα inhibitors have now been widely used in patients with autoimmune diseases and have greatly improved their outcomes. Considering TNFR2’s high expression and its important role in Tregs, it makes targeting TNFR2 a promising immunotherapeutic approach (**Table 1**). However, all these data are from abstracts of AACR Annual Meeting or company media releases, most of these antibodies are still in the early stages of development, and detailed information is unpublished. Aside from the important role that TNFR2 plays in Tregs, TNFR2 is also an oncogene upregulated in certain tumors and can improve cancer cell survival. Therefore, TNFR2 antagonists can block both immunosuppressive cells and certain tumor cells, which have the effect of killing two birds with one stone.

**Table 1:** TNFR2 antibodies under clinical trials or preclinical developments.

	Name	Producer	Stage of development	Condition or disease	Reported mechanisms	References
Antagonist	BI-1808	BioInvent International AB	Phase 1/2a (NCT04752826): monotherapy or combination with anti-PD-1 (Merck)	Human advanced malignancies	a) Intra-tumor Treg depletion b) CD8+ T-cell expansion c) Modulation of tumor-associated myeloid cells	(54, 55)
	BITR2101	Boston Immune Technologies & Therapeutics Inc.	Preclinical development (IND enabling)	Caner (not detailed)	Not detailed	(56)
	APX601	Apexigen Inc.	Preclinical development (IND enabling)	Mouse cancer model (CT26 and MC38)	a) Blockade of the immunosuppressive functions of both Tregs and MDSCs b) Depletion of TNFR2-expressing Tregs, MDSC, and tumor cells *via* ADCC and ADCP c) Tumor growth inhibition both as a single agent and in combination with anti-PD-1	(57, 58)
	AN3025	Adlai Nortye	Preclinical development	Jurkat cell line Mouse cancer model (MC38)	a) Treg depletion b) Increased IFNγ and granzyme expression c) Synergistic effect with anti-PD-1	(59)
	SIM0235	Simcere	Preclinical development (IND enabling)	Cancer (not detailed)	a) Kill TNFR2+ Tregs and MDSCs through ADCC and ADCP b) Kill TNFR2+ tumor cells c) Synergistic effects with anti-PD-L1	(60)
	LBL-019	Nanjing Leads Biolabs Co. Ltd.	Phase 1 (in both China and the USA)	Cancer (not detailed)	Not detailed	(61)
	NBL-020	NovaRock Biotherapeutics	Preclinical development (IND enabling)	Mouse cancer model	a) Enhance CD8 T-cell function through overcoming the suppressive effect from Tregs b) Invigorate exhausted CD8 T cells in an FcγR-dependent manner c) Synergistic effects with anti-PD-L1	(62)
Agonist						
	BI-1910	BioInvent International AB	Preclinical development	Mouse cancer model (CT26, MC38 and B16),	a) Increase CD8+ T cell b) Improved CD8/Treg ratios c) synergistic effects with anti-PD-1	(63)
	HFB200301	HiFiBiO Therapeutics	Preclinical development (IND enabling)	Mouse cancer model (MC38)	a) Activates CD4+, CD8+ T cells, and NK cells *in vitro* b) Expand CD4+T/CD8+T/NK cells in TME without affecting Tregs numbers *in vivo*	(64)
	MM-401	Merrimack Pharmaceuticals, Inc.	Preclinical development	T cells from healthy donors; ovarian cancer ascites samples	a) Upregulation of activation markers and cytokine production of CD4+ and CD8+ T cells from Healthy donors b) Promote ADCC and deplete Tregs in ovarian cancer ascites samples	(65, 66)

IND, investigational new drug; MDSCs, myeloid-derived suppressor cells; ADCC, antibody-dependent cellular cytotoxicity; ADCP, antibody-dependent cellular phagocytosis.

There are several TNFR2 antagonist antibodies that seem to be promising in the clinical transformation, some of them have already undergone clinical study, and others are about to undergo clinical trials. BI-1808 is a fully human IgG1 mAb that targets TNFR2. A phase 1/2a study of BI-1808 as a single agent or in combination with pembrolizumab in subjects with advanced malignancies is now recruiting. Dose escalation and safety will be assessed in a phase 1 study. Evaluation of BI-1808 infusions as a single dose in ovarian cancer, non-small cell lung cancer, and cutaneous T-cell lymphoma (Sézary syndrome and mycosis fungoides) or in combination with pembrolizumab in ovarian cancer and non-small cell lung cancer will be carried out in a phase 2a study. iRECIST is applied for efficacy assessment of targeting TNFR2 alone or in combination therapy (54). The mechanism of BI-1808 was mediated through intra-tumor Treg depletion, CD8+ T-cell expansion, and modulation of tumor-associated myeloid cells. These findings were confirmed in a humanized mouse model (55). BITR2101 from Boston Immune Technologies and Therapeutics is about to conduct phase 1 clinical trials in order to test the effectiveness of the agent alone or in combination with anti-PD-1 antibody tislelizumab (56). APX601 is a rabbit monoclonal antagonist antibody from Apexigen Inc., which has a high binding affinity of TNFR2. It can reverse immune suppression by targeting TNFR2-expressing Tregs and myeloid-derived suppressor cells (MDSCs) and induce the killing of tumor cells (57, 58). AN3025 from Adlai Nortye can significantly inhibit MC38 tumor growth without impaction on body weight through Treg depletion and increased expression of IFNγ and granzyme. In addition, the combined use of AN3025 and PD-1 antibody can achieve a synergistic effect *in vivo* (59). SIM0235 is a mAb that targets and inhibits TNFR2 from Simcere. It is able to kill TNFR2+ Tregs and MDSCs through antibody-dependent cellular cytotoxicity (ADCC), antibody-dependent cellular phagocytosis (ADCP), and other Fc-receptor functions. Meanwhile, it is able to kill TNFR2+ tumor cells directly. SIM0235 also has significant antitumor efficacy and synergistic effects when combined with PD-L1 antibodies (60). LBL-019 from Leads Biolabs is a TNFR2 antagonist aiming at malignant tumors. It is a first-in-class drug targeting TNFR2 that has been approved for a clinical trial in China and has also been recently approved for a clinical trial by the Food and Drug Administration (FDA) according to its official website. Unfortunately, we could not find more detailed information about that (61). NBL-020 from NovaRock Biotherapeutics can block TNFα ligand binding and potently inhibit TNFR2 signaling in the monocytic cells. Moreover, it can enhance CD8 T-cell function to overcome the suppressive effect from Tregs and invigorate exhausted CD8 T cells in an FcγR-dependent manner. The antitumor effects alone or in combination with PD-L1 inhibitors were also confirmed *in vivo*. This NBL-020 is currently at the IND-enabling stage (62).

It is easy to understand the mechanisms of targeting tumors with TNFR2 antagonists, and blocking TNFR2 may have the effect of killing two birds with one stone: boosting antitumor immune responses and directly killing TNFR2 overexpressing tumor cells and tumor mesenchymal cells. However, there are TNFR2 agonist antibodies that also have remarkable antitumor effects *in vivo*.

In addition to BI-1808, BI-1910 from BioInvent International AB is a TNFR2 agonist antibody that is administered in preclinical development. Its potent antitumor efficacy has been demonstrated both as a single agent and in combination with anti-PD-1 through dramatic CD8+ T-cell increases, which results in improved CD8/Treg ratios and tumor regression (63). HFB200301 is a first-in-class agonistic anti-TNFR2 agonist antibody that binds potently and selectively to TNFR2, which demonstrates potent antitumor activity alone and in combination with anti-PD-1. HFB200301 activates CD4+, CD8+ T cells, and NK cells *in vitro* and induces expansion of CD4+ and CD8+ T cells and NK cells in the TME without affecting regulatory T cells numbers *in vivo* (64). Another TNFR2 agonist antibody is MM-401, which shows T-cell co-stimulation and robust antitumor activity and immune memory in a mouse. It can also upregulate activation markers and cytokine production of CD4+ and CD8+ T cells from healthy donors, as well as promote ADCC in an NK cell-mediated *in vitro* assay and deplete Tregs in ovarian cancer ascites samples; all these results are going to be retested in patient-derived xenograft (PDX) mouse models (65, 66).

**Figure 1:**
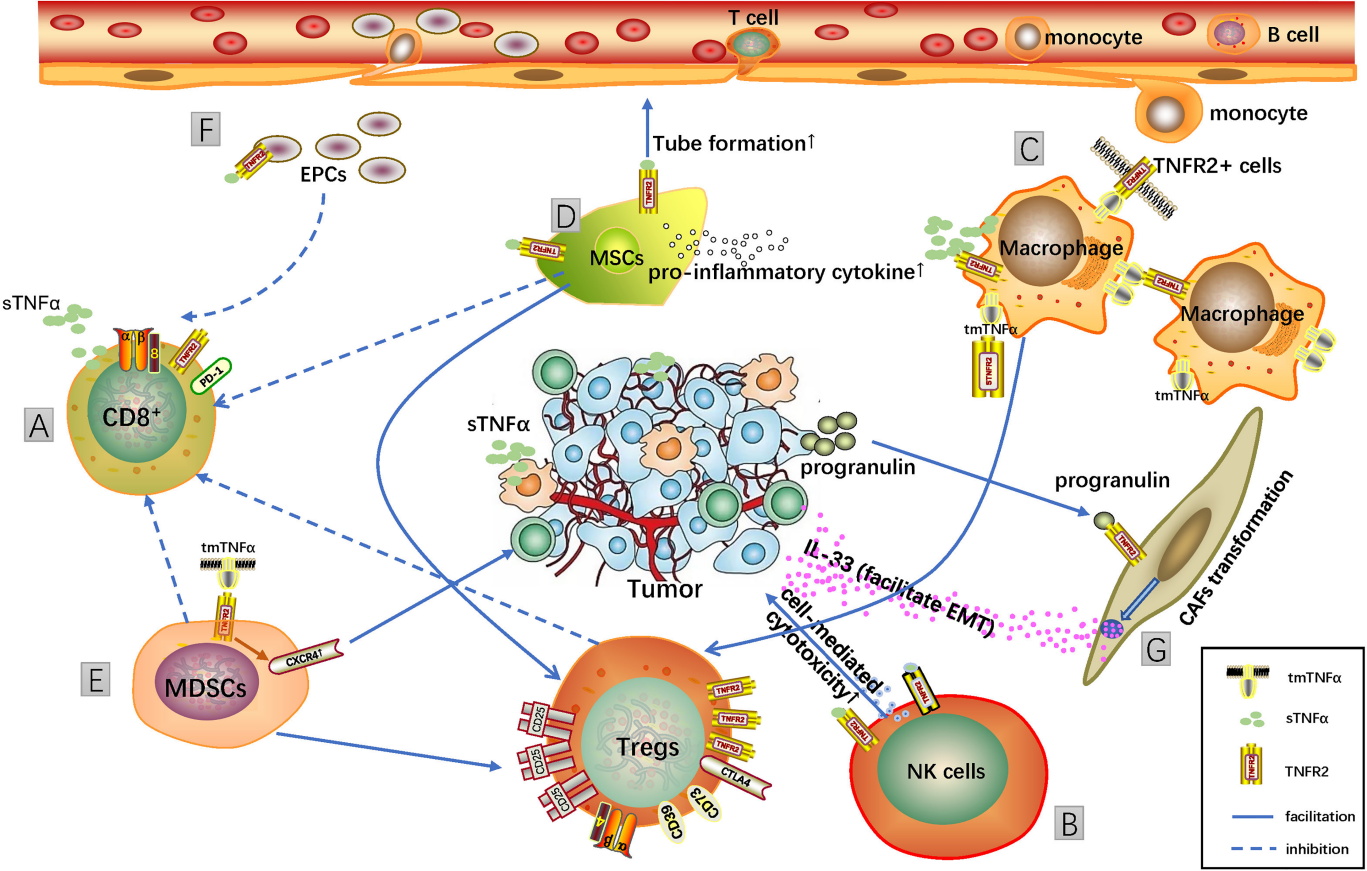
Targeting TNFR2 in different cells in the tumor microenvironment (TME). TME is an indivisible whole complexity, and targeting TNFR2 may influence different cells in it, thus leading to a cascade of reactions and reshaping the immune microenvironment, which achieves antitumor effects ultimately. **(A)** TNFR2 was the predominant TNF receptor on CD8+ effector T cells and required for the secretion of effector molecules and cytotoxic activity of CD8+ T cells. **(B)** Activation of TNF/TNFR2 pathway enhances NK cell cytotoxicity and IFNγ production. **(C)** Macrophages are the major producers of TNFα, and M2 macrophages were more potent in inducing Treg differentiation and proliferation. **(D)** TNFR2+ MSCs can suppress T-cell proliferation, activation, and pro-inflammatory cytokine production and at the same time the induction of active Tregs. **(E)** tmTNFα can induce CXCR4 expression in myeloid-derived suppressor cells (MDSCs) through the TNFR2-dependent pathway, which facilitates the recruitment of MDSCs to tumor tissue with the function of Treg induction and inhibition of T-cell function. **(F)** TNFR2+ EPCs can suppress T-cell proliferation. **(G)** Progranulin can promote the switch from fibroblasts to cancer-associated fibroblasts (CAFs) through the TNFR2 pathway. TNFR2-dependent secretion of IL-33 by CAFs enhances the migration and invasion of cancer cells.

It seems that all roads lead to Rome in either blocking or activating TNFR2 in the tumor. How these two contradictory drugs achieve the same antitumor effect remains to be investigated. TME is an indivisible whole complexity, all the members in this environment may influence each other, and they may react differently in different TNFR2 treatments. Here, we show some typical cell types other than Tregs in TME, which may be involved in targeting TNFR2 (**Figure 1**).

## Non-Malignant Cells Involved in Targeting TNFR2 in TME

### CD8+ T Cells

Previous studies found that TNFR2, but not TNFR1, were the predominant TNF receptor on CD8+ effector T cells (67, 68); the proportion of proliferating transgenic tumor-specific CD8+ T cells in TNFR2 deficient mice was significantly reduced in TDLNs (67). TNFR2 is also required for the secretion of effector molecules and cytotoxic activity of CD8+ T cells (69). Some CD8+ T cells can also secrete cytokines, which include TNFα (70), and TNFα could influence other TNFR+ cells.

A mouse TNFR2 agonist antibody Y9 was identified and had antitumor effects in mouse models, and the effects were mediated by CD8+ T cells and NK cells. TNFR2 agonist treatment could downregulate TNFR2 on T cells, thus leading to CD8+ T-cell expansion and function improvement. However, this agonist did not deplete Tregs. Meanwhile, they generated a parallel anti-human TNFR2 antibody Ab1, which exhibits similar properties to the Y9 antibody, and it can increase proliferation, activation markers, and cytokines in both CD4+ and CD8+ T cells. Moreover, it also has antitumor activity in humanized mouse models (71). This result broke the initial thinking that targeting TNFR2 in cancer only means blocking, and it shed light on the potential possibility of TNFR2 agonist antibodies as antitumor agents in preclinical development.

### Natural Killer Cells

TNFR2 plays a vital role in the function maintenance of NK cells. NK cells play a central role in the antitumor process in TME (2), acting directly through cell-mediated cytotoxicity and by secreting cytokines. Activation of the TNF/TNFR2 pathway enhances NK cell cytotoxicity, and TNFα also enhanced murine NK cell IFNγ production *via* TNFR2 *in vivo* and *in vitro* (72). DC TNFα and NK cell TNFR2 are required for DC-mediated NK cell proliferation and amplification of cytotoxic activity (10).

### Macrophages

Macrophages are the major producers of TNFα and interestingly are also highly responsive to TNFα through TNFR1 and TNFR2 (73). TNFR2-positive tumor-associated macrophages were related to the metastasis of human triple-negative breast cancer (74). tmTNFα can act as a receptor when interacting with sTNFR2- or TNFR2-expressing cells. This reverse signaling is proven to be profoundly important in the activation of monocytes (15, 75). The reverse signaling activated by mTNFα could increase the production of TNFα (75). tmTNFα+ M2 macrophages were more potent in inducing Treg differentiation and proliferation (48). 14-3-3ϵ was essential for TNFR2 signaling-mediated regulation of macrophage polarization and switch (76).

### Mesenchymal Stem Cells

Mesenchymal stem cells (MSCs) have the ability to modulate the immune response and belong to immunosuppressive cells. TNFR2 is a key regulator strongly involved in the immunosuppressive properties of MSCs. This includes suppression of T-cell proliferation, activation, and pro-inflammatory cytokine production and at the same time the induction of active Tregs (77). TNFR2 expression by MSCs is also associated with enhanced tube formation property. TNFR2 plays a critical role in controlling MSC biological and functional properties (78, 79).

### Myeloid-Derived Suppressor Cells

MDSCs are well known for their capacity of promoting immune evasion in tumor sites. tmTNFα can induce CXCR4 expression in MDSCs through the TNFR2-dependent pathway, which facilitates the recruitment of MDSCs to tumor tissue. CXCR4 inhibitor could impair the MDSC accumulation in tumors of TNFR^−/−^ mice after the restoration of adoptive transfer of wild-type MDSCs (80). So tmTNFα acts as a potent activator of MDSCs *via* the TNFR2 pathway and promotes tumor immune escape (81). Moreover, the ability of MDSCs to induce Tregs *in vivo* has been described (82, 83), and they can also inhibit T-cell function in a non-specific manner (84).

### Endothelial Cells and EPCs

Endothelial progenitor cells (EPCs) are non-differentiated endothelial cells (ECs). They are involved in cancer-associated neo-vascularization, thus facilitating cancer progression (85). Evidence showed that EPCs were able to suppress T-cell proliferation, and the TNFα/TNFR2 signaling pathway in EPCs played a key regulatory factor in this immunosuppressive effect (86). Adequate TNFα preconditioning could increase TNFR2 expression without an unrestrained increase of TNFR1 and prime EPCs towards more immunosuppressive functions (87).

### Cancer-Associated Fibroblasts

Cancer-associated fibroblasts (CAFs) are the activated fibroblasts in cancer stroma that can promote cancer progression by the secretion of cytokines and interaction with the local extracellular matrix. In gastric cancer, CAF-derived IL-33 enhances the migration and invasion of gastric cancer cells by inducing the epithelial–mesenchymal transition (EMT), and the secretion of IL-33 by CAFs is dependent on the activation of the TNFR2-NF-κB-IRF1 pathway (88). Progranulin secreted by colorectal cancer cells can promote the switch from fibroblasts to CAFs through the TNFR2 pathway (89).

## TNFR2 in Tumor Cells

Besides immune and mesenchymal cells, increased TNFR2 expression has also been found in several types of tumors, such as ovarian cancer, colon cancer, kidney cancer, and T-cell lymphomas (39, 90–93). It seems that hematopoietic and lymphoid cells have the highest expression of TNFR2 in 788 human tumor cell lines (93), which indicated their vital role in the maintenance of tumor cell vitality. In the retrospective studies, TNFR2 expression is higher in tumor sites than non-tumor sites in esophageal cancer, and TNFR2 is positively correlated with high malignancy and poorer survival (94). Similar results have been obtained in non-small cell lung cancer and ovarian cancer (95, 96).

## Discussion

TNFR2 has emerged as a potential immune checkpoint in cancer treatment; however, the role it played in TME is much more complex than we thought. The antitumor effects of targeting TNFR2 can be concluded as direct inhibition of cell proliferation and influence immune cells and then kill tumor cells indirectly. TNFR2 antagonist antibodies can inhibit TNFR2-positive cancer cells and tumor supporting cells, such as CAFs, ECs, and EPCs, directly by signal interference. TNFR2 antibodies could also attenuate the function of immunosuppressive cells or enhance the killing ability of effector T cells directly to achieve antitumor effects. A new mechanism needs to be deployed considering the complicated network of TME. Meanwhile, we are looking forward to the results of these clinical trials and hoping targeting TNFR2 may achieve huge success in immunotherapy and benefit more tumor patients.

## Author Contributions

JB was responsible for the data collection and the draft of the manuscript. BD gave the necessary assistance to finish the manuscript. HL designed the project and modified the paper.

## Funding

This study was funded by the National Natural Science Foundation of China (grant number 82171728).

## Conflict of Interest

The authors declare that the research was conducted in the absence of any commercial or financial relationships that could be construed as a potential conflict of interest.

## Publisher’s Note

All claims expressed in this article are solely those of the authors and do not necessarily represent those of their affiliated organizations, or those of the publisher, the editors and the reviewers. Any product that may be evaluated in this article, or claim that may be made by its manufacturer, is not guaranteed or endorsed by the publisher.
